# Association Between Dietary Copper Intake and Incident Metabolic Syndrome in Chinese Adults: A Discrete-Time Hazard Model Analysis

**DOI:** 10.3390/nu18101539

**Published:** 2026-05-12

**Authors:** Zhongting Lu, Zhihong Wang, Jiguo Zhang, Lixin Hao, Boya Zhao, Liusen Wang, Huijun Wang, Hongru Jiang

**Affiliations:** 1 National Institute for Nutrition and Health, Chinese Center for Disease Control and Prevention, Beijing 100050, China; 202216405@mail.sdu.edu.cn (Z.L.); wangzh@ninh.chinacdc.cn (Z.W.); zhangjg@ninh.chinacdc.cn (J.Z.); haolx@ninh.chinacdc.cn (L.H.); zhaoby@ninh.chinacdc.cn (B.Z.); wangls@ninh.chinacdc.cn (L.W.); wanghj@ninh.chinacdc.cn (H.W.); 2 Key Laboratory of Public Nutrition and Health, National Health Commission of the People’s Republic of China, Beijing 100050, China; 3 Key Laboratory of Food Nutrition and Health Evaluation Technology, State Administration for Market Regulation, Beijing 100050, China

**Keywords:** dietary copper intake, metabolic syndrome, discrete-time hazard model, China Health and Nutrition Survey

## Abstract

Objective: The aim of this study was to examine the association between dietary copper intake and incident metabolic syndrome (MetS) in Chinese adults. Methods: Data were obtained from the China Health and Nutrition Survey (CHNS) in 2009, 2015, and 2018. A total of 2418 adults aged 18–64 years who were free of MetS at baseline in 2009 were included. Person-period data were constructed, and discrete-time hazard models with a complementary log-log link were used to evaluate the associations of dietary copper intake with incident MetS and its components. Restricted cubic spline analysis (RCS) was used to assess the dose–response relationship. Results: After multivariable adjustment, compared with the lowest quartile (Q1) of dietary copper intake, the Q4 groups had a higher risks of incident MetS, with HR (95% CI) of 1.32 (1.06–1.65) (*p* = 0.014). When analyzed as a continuous variable, each 1 mg/1000 kcal increase in energy-adjusted dietary copper intake was associated with an approximately 27% higher risk of incident MetS (HR = 1.27, 95% CI: 1.03–1.53) (*p* = 0.023). RCS indicated a significant overall association without evidence of nonlinearity. Component analyses suggested that higher dietary copper intake might be associated with increased risks of reduced high-density lipoprotein cholesterol (HDL-C) and abdominal obesity. Sensitivity analyses were generally consistent with the main findings. Conclusions: Higher dietary copper intake was associated with an increased risk of incident MetS in Chinese adults. Component analyses suggested a more consistent association for reduced HDL-C, while an additional possible association was observed for abdominal obesity.

## 1. Introduction

Metabolic syndrome (MetS) is a cluster of metabolic abnormalities characterized by central obesity, insulin resistance, dyslipidemia, and elevated blood pressure, and is associated with substantially increased risks of cardiovascular disease, type 2 diabetes, and all-cause mortality [1,2]. With the rapid socioeconomic transition and lifestyle changes in China, the prevalence of MetS has risen markedly [3,4,5], making it a major public health challenge.

Dietary nutrients play a critical role in the development and progression of MetS [6,7]. Copper is an essential trace element in humans and serves as a cofactor for multiple metalloenzymes, including cytochrome c oxidase, superoxide dismutase, and lysyl oxidase. It is involved in a wide range of physiological processes, such as mitochondrial energy metabolism, antioxidant defense, glucose homeostasis, and lipid metabolism [8,9]. Adequate copper intake is important for maintaining glucose homeostasis and lipoprotein metabolism [10,11]. However, imbalanced copper intake, particularly long-term high intake, may promote metabolic disturbances through mechanisms involving oxidative stress, mitochondrial dysfunction, impaired autophagy, and alterations in signaling pathways such as Nrf2 and PPARγ, thereby contributing lipid accumulation and dysregulated lipid metabolism [12,13,14,15].

Epidemiological evidence regarding the association of dietary copper intake with MetS and its components remains inconsistent. A meta-analysis of observational studies suggested that higher dietary copper intake may be inversely associated with MetS risk; however, most included studies were cross-sectional, and prospective evidence remains very limited [16]. For example, a cross-sectional study conducted in Harbin, China, reported an inverse association between higher dietary copper intake and MetS risk (OR ≈ 0.81, 90% CI: 0.70–0.94; *p* for trend = 0.020) [17]. In contrast, studies based on biomarkers have tended to suggest that a higher internal copper burden may be associated with adverse metabolic phenotypes, although the findings were not entirely consistent. A nested case–control study with 3 years of follow-up among middle-aged and older Chinese adults did not observe significant associations between serum copper levels and the risk of incident MetS or its components [18]. By contrast, a case–control study in Taiwanese adults found that higher serum copper levels were positively associated with MetS risk, with an OR of 2.02 (95% CI: 1.25–3.25; *p* for trend = 0.013) for the highest versus lowest quartile [19]. In addition, another study reported that, among overweight or obese adults, higher dietary copper intake was positively associated with insulin resistance [20].

Overall, the current epidemiological evidence on the relationship between copper and MetS remains inconclusive and has largely been derived from cross-sectional studies, with relatively limited prospective evidence. In particular, longitudinal evidence on the association between dietary copper intake and the risk of incident MetS among Chinese adults remains scarce. Therefore, using data from multiple waves of the China Health and Nutrition Survey (CHNS), this study applied a discrete-time survival analysis to systematically examine the association between dietary copper intake and the risk of incident MetS, with the aim of providing new evidence to inform dietary prevention strategies for MetS.

## 2. Materials and Methods

### 2.1. Study Population

This study used data from the CHNS, an ongoing prospective cohort jointly conducted by the National Institute for Nutrition and Health, Chinese Center for Disease Control and Prevention, and the Carolina Population Center at the University of North Carolina at Chapel Hill. The CHNS used a multistage, stratified, cluster sampling design and collected repeated information on demographic characteristics, dietary intake, lifestyle factors, and health status from residents across multiple provinces in China. For the present analysis, we used data from the 2009, 2015, and 2018 survey waves. Participants aged 18–64 years who were free of MetS in 2009 and had complete information on MetS ascertainment, anthropometric measurements, blood biochemical tests, and dietary assessment were eligible. We excluded participants who were pregnant, had implausible total energy intake (<500 or >5000 kcal/day). A total of 2418 participants were included in the main analysis. To further examine the associations between dietary copper intake and the individual components of MetS, we additionally constructed five component-specific analytic cohorts. For each component, participants free of that specific abnormality at baseline were included according to the same eligibility criteria. The study protocol was approved by the Ethics Committee of the National Institute for Nutrition and Health, Chinese Center for Disease Control and Prevention (approval number: No. 2018-004, approval date: 14 March 2018), and all participants provided written informed consent after receiving a full explanation of the study.

### 2.2. Definition of Metabolic Syndrome

MetS was defined according to the 2009 Joint Interim Statement for harmonizing the metabolic syndrome [21]. Participants were classified as having MetS if they met at least three of the following five criteria: (1) elevated blood pressure, defined as systolic blood pressure ≥ 130 mmHg, diastolic blood pressure ≥ 85 mmHg, or current use of antihypertensive medication; (2) elevated fasting plasma glucose (FPG), defined as FPG ≥ 5.6 mmol/L or current use of glucose-lowering medication; (3) elevated triglycerides (TG), defined as TG ≥ 1.70 mmol/L; (4) reduced high-density lipoprotein cholesterol (HDL-C), defined as HDL-C < 1.04 mmol/L for men or < 1.30 mmol/L for women; and (5) abdominal obesity, defined as waist circumference ≥ 90 cm for men or ≥80 cm for women.

### 2.3. Assessment of Dietary Copper Intake and Covariates

Dietary intake over the previous year was assessed using a validated food frequency questionnaire (FFQ), which covered 65 commonly consumed food items across nine food categories [22]. Information was collected on whether each food item was consumed, consumption frequency, and usual amount consumed per occasion, and food intakes were converted into average daily intake. Based on the Chinese Food Composition Table [23], foods included in the FFQ were grouped into predefined food groups. To reduce within-group heterogeneity in nutrient estimation, foods were further standardized using subgroup-specific weighting coefficients derived from the Scientific Evidence and Methodology of the Dietary Guidelines [24]. On this basis, average daily energy intake and dietary copper intake were calculated for each participant using the food composition database. To account for variation in total energy intake, dietary copper intake was further expressed as an energy-adjusted nutrient density, calculated as copper intake per 1000 kcal of total energy intake (mg/1000 kcal), and this measure was used as the primary exposure in the main analysis.

Covariates were collected through standardized face-to-face interviews conducted by trained investigators. Age, body mass index (BMI), sleep duration, sedentary time, physical activity, and total energy intake were treated as continuous variables. Gender, smoking status, alcohol drinking status, education level, income level, and residence were treated as categorical variables. Household annual income was categorized into low, medium, and high groups according to tertiles. Education level was classified as primary school or below, secondary school, and college or above. Residence was classified as urban or rural.

### 2.4. Construction of Person-Period Data

Because the exact date of MetS onset was unavailable and incident events could only be ascertained between two consecutive survey waves, the outcome was analyzed as discrete-time survival data. The cohort was restructured into a person-period dataset, in which each participant contributed one record for each follow-up interval during which he or she remained at risk [25]. The binary outcome for each record indicated whether incident MetS had occurred by the end of that interval. Participants contributed records until the occurrence of incident MetS, loss to follow-up, or the end of follow-up. A total of 3634 person-period records were generated, including 2418 records for the 2009–2015 interval and 1216 records for the 2015–2018 interval. Dietary copper intake and selected interval-updated covariates were assigned using values measured at the beginning of each interval. Quartiles of energy-adjusted dietary copper intake (mg/1000 kcal) were defined based on the pooled distribution of interval-specific baseline values from the 2009–2015 and 2015–2018 intervals, and participants were categorized into quartiles (Q1–Q4) accordingly.

### 2.5. Statistical Analysis

Discrete-time hazard models with a complementary log-log link were used to estimate the associations between dietary copper intake and incident MetS. A time-factor variable representing follow-up interval was included in all models to account for differences in baseline hazard across intervals. To assess whether the association between dietary copper intake and incident MetS varied across follow-up intervals, interaction terms between copper intake quartiles and the time-interval indicator were additionally tested. As no significant interaction was observed, pooled estimates are presented. Under the complementary log-log specification, exponentiated coefficients were interpreted approximately as hazard ratios (HRs).

Three progressively adjusted models were fitted. Model 1 included only the time-interval indicator. Model 2 further adjusted for age, gender, residence, education level, income level, smoking status, alcohol drinking status, and physical activity. Model 3 additionally adjusted for sleep duration, sedentary time, BMI, and total energy intake. We further examined associations between dietary copper intake and the five individual components of MetS using separate discrete-time hazard models in the corresponding component-specific cohorts. For elevated FPG, elevated TG, and reduced HDL-C, covariate adjustment was the same as in the main analysis. For elevated blood pressure, dietary sodium intake was additionally included in Model 3 because of its established relevance to blood pressure. For abdominal obesity, BMI was not included in Model 3 to avoid overadjustment due to its close correlation with central adiposity. Restricted cubic spline (RCS) analysis was performed to explore the dose–response relationship between dietary copper intake and incident MetS. Four knots were placed at the 5th, 35th, 65th, and 95th percentiles of dietary copper intake, with the 12.5th percentile used as the reference value. All statistical analyses were conducted using R software, version 4.5.2. All tests were two-sided, and *p* < 0.05 was considered statistically significant.

### 2.6. Sensitivity Analysis Method

In a sensitivity analysis, BMI and total energy intake were fixed at their 2009 baseline values, whereas all other interval-updated covariates were updated using values measured at the beginning of each observation interval, to assess the robustness of the findings to alternative handling of these covariates.

## 3. Results

### 3.1. Baseline Characteristics of Participants

A total of 2418 participants were included, comprising 875 men (36.2%) and 1543 women (63.8%). The overall median energy-adjusted dietary copper intake was 0.85 mg/1000 kcal (IQR: 0.72–1.03), and the overall median absolute copper intake was 1.84 mg/d (IQR: 1.45–2.40). Across Q1 to Q4 of energy-adjusted copper intake, the median energy-adjusted intakes were 0.63, 0.77, 0.91, and 1.18 mg/1000 kcal, respectively. The overall incidence of MetS was 28.7% (695/2418), with corresponding incidences of 29.2%, 30.5%, 25.9%, and 29.5% from Q1 to Q4, respectively; the difference was not statistically significant (*p* = 0.296). The distributions of gender, education level, smoking status, BMI, physical activity, and total energy intake differed significantly across quartiles (all *p* < 0.05). By contrast, residence, income level, alcohol drinking status, age, sleep duration, and sedentary time did not differ significantly across quartiles (all *p* > 0.05) (Table 1).

### 3.2. Major Food Sources of Dietary Copper Intake at Baseline and Follow-Up

**Figure 1:**
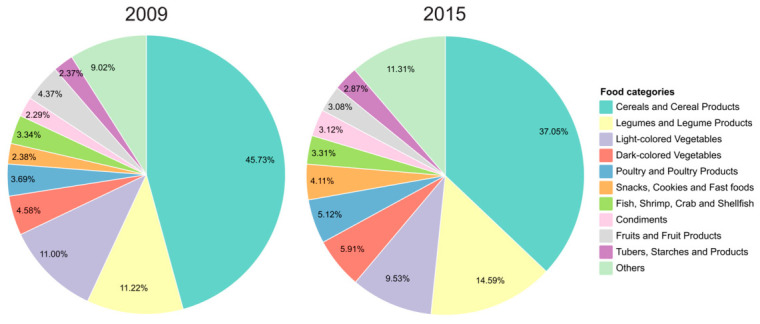
Major food sources of dietary copper intake among study participants at Baseline and Follow-Up.

To further describe the food sources of dietary copper intake, we summarized the major contributors at the start of each observation interval. Specifically, dietary copper sources in 2009 were summarized among the 2418 participants in the initial risk set for the 2009–2015 interval, whereas dietary copper sources in 2015 were described for the 1216 participants who remained at risk and entered the 2015–2018 interval. At both time points, dietary copper intake was mainly derived from cereals and cereal products, legumes and legume products, and vegetables. Cereals and cereal products remained the largest contributor at both time points, accounting for 45.73% in 2009 and 37.05% in 2015. Legumes and legume products accounted for 11.22% in 2009 and 14.59% in 2015. The proportional contributions of dark-colored vegetables, poultry and poultry products, snacks/cookies/fast foods, and condiments were also higher in 2015 than in 2009 (Figure 1).

### 3.3. Association Between Energy-Adjusted Dietary Copper Intake and Incident MetS

**Figure 2:**
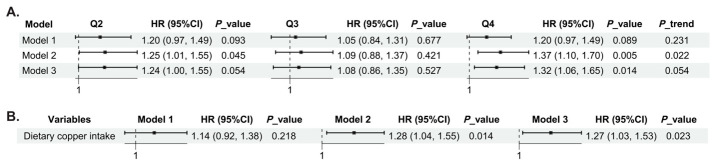
Associations between energy-adjusted dietary copper intake and the risk of incident MetS. (**A**) Energy-adjusted dietary copper intake analyzed as a categorical variable, with Q1 as the reference group. (**B**) Energy-adjusted dietary copper intake analyzed as a continuous variable. Model 1 was adjusted for the time-interval indicator only. Model 2 was further adjusted for age, gender, residence, education level, income level, smoking status, alcohol drinking status, and physical activity. Model 3 was additionally adjusted for sleep duration, sedentary time, BMI, and total energy intake.

**Figure 3:**
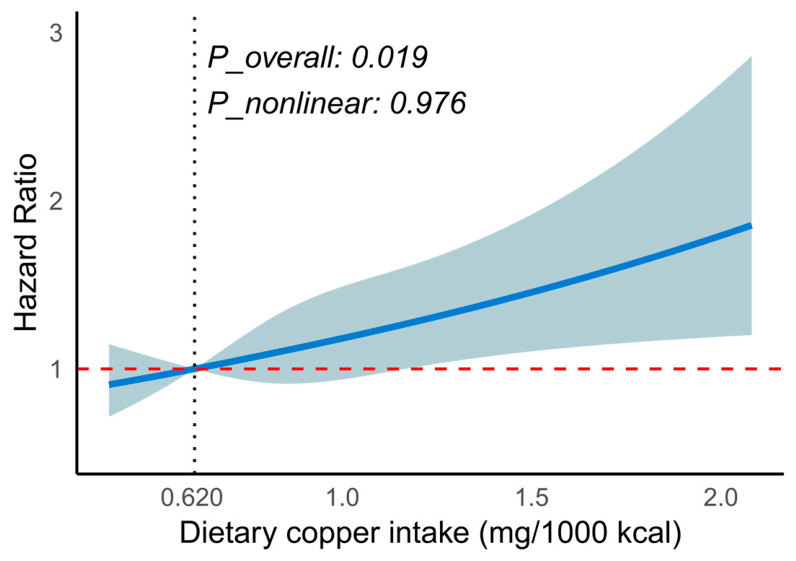
Restricted cubic spline curve for the association between energy-adjusted dietary copper intake and the risk of incident MetS. The curve was estimated using the fully adjusted model (Model 3), with adjustment for the time-interval indicator, age, gender, residence, education level, income level, smoking status, alcohol drinking status, physical activity, sleep duration, sedentary time, BMI, and total energy intake. The solid line represents the estimated HR, and the shaded area represents the 95% CI. The horizontal reference line indicates HR = 1. The reference value was set at the 12.5th percentile of energy-adjusted dietary copper intake.

After multivariable adjustment, Q4 of energy-adjusted dietary copper intake was associated with a higher risk of incident MetS compared with Q1 (Model 3: HR = 1.32, 95% CI: 1.06–1.65, *p* = 0.014). The trend across quartiles was borderline but did not reach conventional statistical significance (*p* for trend = 0.054) (Figure 2A). When energy-adjusted dietary copper intake was analyzed as a continuous variable, each 1 mg/1000 kcal increment was associated with an approximately 27% higher risk of incident MetS in Model 3 (HR = 1.27, 95% CI: 1.03–1.53, *p* = 0.023) (Figure 2B). Restricted cubic spline analysis further indicated a significant overall association between energy-adjusted dietary copper intake and incident MetS (*p*-overall = 0.019), with no evidence of nonlinearity (*p*-nonlinear = 0.976) (Figure 3).

After further including interaction terms between dietary copper intake quartiles and time-interval indicator, the likelihood ratio test showed no significant improvement in model fit (*p* for interaction = 0.313). In addition, none of the individual interaction terms was statistically significant (Q2: *p* = 0.131; Q3: *p* = 0.253; Q4: *p* = 0.929), suggesting no substantial heterogeneity in the association between dietary copper intake and incident MetS across the two follow-up intervals (2009–2015 and 2015–2018).

### 3.4. Associations Between Energy-Adjusted Dietary Copper Intake and Individual Components of MetS

Among the individual components of MetS, the incidences of elevated blood pressure, elevated FPG, elevated TG, reduced HDL-C, and abdominal obesity were 49.9% (961/1925), 35.7% (833/2335), 27.0% (552/2044), 27.0% (594/2204), and 29.5% (599/2030), respectively. In unadjusted comparisons, the incidence proportions of all five components did not differ significantly across quartiles (all *p* > 0.05) (Table 2).

**Figure 4:**
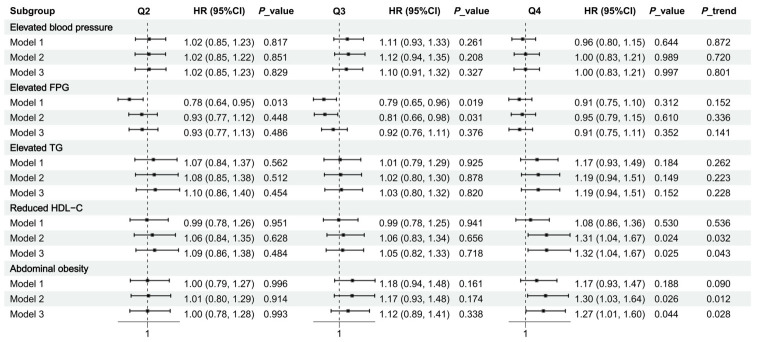
Associations of quartiles of energy-adjusted dietary copper intake with the risks of incident individual components of metabolic syndrome. Q1 was used as the reference group. Separate discrete-time hazard models were fitted for each MetS component using the corresponding component-specific cohort, including only participants free of that abnormality at baseline. Model 1 was adjusted for the time-interval indicator only. For elevated FPG, elevated TG, and reduced HDL-C, Model 2 was further adjusted for age, gender, residence, education level, income level, smoking status, alcohol drinking status, and physical activity; Model 3 was additionally adjusted for sleep duration, sedentary time, BMI, and total energy intake. For elevated blood pressure, dietary sodium intake was additionally included in Model 3. For abdominal obesity, Model 3 included the same covariates as the main Model 3 except BMI.

In multivariable analyses, dietary copper intake was not significantly associated with the risks of incident elevated blood pressure, elevated FPG, or elevated TG. By contrast, in the fully adjusted model, Q4 was associated with a higher risk of reduced HDL-C than Q1 [HR (95% CI) = 1.32 (1.04–1.67), *p* = 0.025], with a significant trend across quartiles (*p* for trend = 0.043). For abdominal obesity, a similar pattern was observed in Model 3 without BMI adjustment [HR (95% CI) = 1.27 (1.01–1.60), *p* = 0.044; *p* for trend = 0.028] (Figure 4).

### 3.5. Analyses Accounting for Major Dietary Sources of Copper

**Figure 5:**
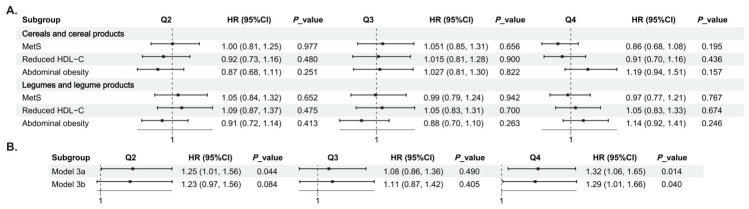
Analyses accounting for major dietary sources of copper. (**A**) Associations of cereals and cereal products as well as legumes and legume products with incident MetS, reduced HDL-C, and abdominal obesity. (**B**) Associations between energy-adjusted dietary copper intake and incident MetS after additional adjustment for cereals and cereal products alone (Model 3a) and for both food groups (Model 3b). All models were based on the fully adjusted model; BMI was not included in models for abdominal obesity.

To evaluate whether the observed association between energy-adjusted dietary copper intake and incident MetS was driven by major dietary sources of copper, we focused on cereals and cereal products as well as legumes and legume products. In fully adjusted models, neither food group was significantly associated with incident MetS, reduced HDL-C, or abdominal obesity (all *p* > 0.05) (Figure 5A). We further adjusted the main model for cereals and cereal products alone (Model 3a), and subsequently for both food groups (Model 3b). The association between energy-adjusted dietary copper intake and incident MetS remained generally consistent; the HRs (95% CIs) for Q4 versus Q1 were 1.32 (1.06–1.65) in Model 3a and 1.29 (1.01–1.66) in Model 3b (both *p* < 0.05) (Figure 5B).

### 3.6. Sensitivity Analysis

**Figure 6:**
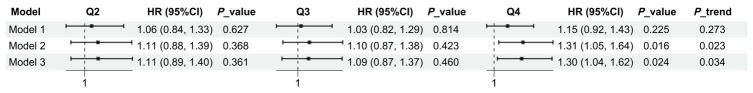
Associations between quartiles of energy-adjusted dietary copper intake and the risk of incident MetS in the sensitivity analysis. Q1 was used as the reference group. Model 1 was adjusted for the time-interval indicator only. Model 2 was further adjusted for age, gender, residence, education level, income level, smoking status, alcohol drinking status, and physical activity. Model 3 was additionally adjusted for sleep duration, sedentary time, BMI, and total energy intake. In the sensitivity analysis, BMI and total energy intake were included as baseline-fixed covariates at their 2009 baseline values, whereas all other covariates were updated at the beginning of each observation interval.

In the sensitivity analysis, the positive association between higher energy-adjusted dietary copper intake and the risk of incident MetS remained after fixing BMI and total energy intake at their 2009 baseline values while updating all other covariates at the beginning of each observation interval. In Model 3, participants in Q4 had a higher risk of incident MetS than those in Q1 (HR = 1.30, 95% CI: 1.04–1.62; *p* = 0.024), with a significant trend across quartiles (*p* for trend = 0.034) (Figure 6).

## 4. Discussion

Using longitudinal repeated-measures data from the CHNS and discrete-time hazard models based on observation intervals, we found that higher dietary copper intake was associated with a higher risk of incident MetS after multivariable adjustment. RCS analysis indicated a significant overall association but no evidence of nonlinearity, suggesting an approximately linear positive association across the observed range of intake. In the component analyses, the association was most evident for reduced HDL-C. An additional association was observed for abdominal obesity; however, this finding was derived from the model without BMI adjustment and should be interpreted cautiously.

Our findings differ from some previous cross-sectional studies suggesting an inverse association between dietary copper intake and MetS [16]. A likely explanation is the difference in study design. In contrast to cross-sectional analyses, our longitudinal design used incident MetS as the outcome and provided a clearer temporal sequence, thereby reducing the likelihood of reverse causation. It is also noteworthy that the crude incidence of MetS did not differ significantly across quartiles of dietary copper intake, whereas the multivariable models showed a higher risk for Q4 in Model 3. This pattern may indicate that the crude incidence reflected the combined influence of heterogeneous demographic, behavioral, and dietary characteristics across copper intake groups rather than the adjusted association of dietary copper intake itself. After covariate adjustment, the association became more apparent. The sensitivity analysis, in which BMI and total energy intake were fixed at baseline values, yielded results in the same direction, although with slightly attenuated effect estimates, supporting the robustness of the main findings while also indicating that the handling of these covariates may influence effect size.

The discrepancy with earlier cross-sectional evidence may also relate to differences in background copper nutrition. The recommended nutrient intake for copper in Chinese adults is 0.8 mg/d [26]. In our study, even the lowest intake group had a median absolute intake of 1.33 mg/d, suggesting that the study population was unlikely to be characterized by low copper intake. Thus, the observed association should not be interpreted as reflecting benefits from correcting copper inadequacy. Because dietary copper is embedded in habitual food consumption, we further examined whether the association was attributable to major copper-contributing food groups. Neither cereals and cereal products nor legumes and legume products were significantly associated with incident MetS, reduced HDL-C, or abdominal obesity, and additional adjustment for these food groups did not materially change the copper–MetS association. These findings suggest that the association was not fully explained by the major dietary sources considered, although residual confounding by other dietary constituents or overall dietary patterns cannot be excluded. Importantly, intake above the recommended level should not be equated with toxicity or harmful excess. A more cautious interpretation is that, in a population without apparent copper inadequacy, higher copper intake may not necessarily be metabolically advantageous [27]. This interpretation is consistent with emerging evidence from Chinese populations suggesting that higher copper intake is not uniformly associated with better health outcomes, including a U-shaped association with abdominal obesity in CHNS participants [28], a J-shaped association with all-cause mortality in Chinese adults [29], and a positive association of higher dietary copper intake with cardiovascular events and mortality in the PURE-China study [30].

Although our findings at the dietary level were not fully aligned with some cross-sectional studies of MetS, they were directionally closer to biomarker-based evidence suggesting that higher internal copper burden may be related to adverse metabolic phenotypes. For example, a case–control study in Taiwanese adults reported that higher serum copper levels were significantly associated with an increased risk of MetS [19]. In addition, among women with obesity, copper-related biomarkers were positively associated with insulin resistance parameters and dyslipidemia [31]. Data from the National Health and Nutrition Examination Survey also suggested that higher serum copper levels were associated with an increased risk of obesity [32]. Copper absorption, transport, utilization, and excretion are tightly regulated by intestinal uptake, hepatic handling, ceruloplasmin binding, and copper transport systems such as ATP7A and ATP7B [8]. Under physiological conditions, this homeostatic network can buffer a certain range of dietary variation [33]. However, persistently higher dietary exposure may be associated with altered copper handling or redox balance, although this could not be evaluated directly in the present study [34]. In the absence of biomarkers such as serum copper, urinary copper, or ceruloplasmin, our study cannot directly determine whether higher dietary intake translated into higher internal copper burden [35]. Nevertheless, in a population with generally adequate or relatively high copper intake, long-term higher dietary exposure may be associated with a less favorable metabolic profile. This may help explain why our findings were more consistent with biomarker-based studies than with some cross-sectional dietary analyses.

By examining incident risk for each MetS component separately, this study further suggested that reduced HDL-C may be a more sensitive phenotype in relation to dietary copper intake. Although the crude incidence proportions of all five components did not differ significantly across quartiles, some associations emerged after multivariable adjustment, particularly for reduced HDL-C. For reduced HDL-C, the association remained significant after adjustment. For abdominal obesity, the association was observed in the model without BMI adjustment; therefore, it should be regarded as a secondary finding rather than evidence of the same strength as that for reduced HDL-C.

Several limitations should be considered. First, the exact timing of MetS onset was unavailable and could only be localized to the interval between two survey waves. A discrete-time hazard model is appropriate for this type of interval-defined outcome; however, it cannot determine whether an event occurred early or late within a given interval. In addition, the two follow-up intervals were of unequal duration. To account for this, we included a time-interval indicator and examined the interaction between dietary copper intake quartiles and time-interval indicator. Neither the overall interaction test nor the individual interaction terms was statistically significant, providing no clear evidence that the association between dietary copper intake and incident MetS differed materially between the 2009–2015 and 2015–2018 follow-up intervals. Nevertheless, more granular temporal changes in exposure, covariates, and event timing could not be captured. Second, slightly different covariate adjustment strategies were used across component-specific models, and effect sizes should therefore not be compared directly across components. Third, dietary copper intake was estimated from dietary survey data and remained susceptible to recall bias and measurement error. Fourth,, residual confounding from other dietary constituents, cooking practices, and unmeasured lifestyle factors cannot be excluded. Finally, no biomarkers of copper status were available, precluding further distinction between dietary exposure and internal copper burden. Future studies integrating dietary assessment with biomarkers such as blood copper, ceruloplasmin, and copper transport proteins will be important for clarifying the metabolic relevance of copper exposure.

## 5. Conclusions

In this cohort of Chinese adults, higher dietary copper intake was associated with a higher risk of incident MetS after multivariable adjustment. This association was most evident for reduced HDL-C, with an additional signal observed for abdominal obesity. Given the observational nature of the study, these findings should be interpreted cautiously and warrant further confirmation in studies incorporating biomarkers of copper metabolism and, where feasible, intervention-based designs.

## Figures and Tables

**Table 1:** Baseline characteristics of the participants in 2009 according to quartiles of energy-adjusted dietary copper intake density.

Variables	Total	Q1	Q2	Q3	Q4	p_Value
Energy-adjusted dietary copper intake, mg/1000 kcal, Median (IQR)	0.85 (0.72, 1.03)	0.63 (0.56, 0.67)	0.77 (0.73, 0.8)	0.91 (0.87, 0.96)	1.18 (1.09, 1.35)	—
Absolute dietary copper intake, mg/d, Median (IQR)	1.84 (1.45, 2.40)	1.33 (1.10, 1.65)	1.72 (1.42, 2.06)	1.92 (1.63, 2.36)	2.64 (2.05, 3.25)	—
Incident MetS, *n/N* (%)	695/2418 (28.7)	155/530 (29.2)	186/610 (30.5)	166/641 (25.9)	188/637 (29.5)	0.296
Gender, *n* (%)						<0.001
Male	875 (36.2)	225 (42.5)	236 (38.7)	227 (35.4)	187 (29.4)	
Female	1543 (63.8)	305 (57.5)	374 (61.3)	414 (64.6)	450 (70.6)	
Residence, *n* (%)						0.637
Urban	641 (26.5)	146 (27.5)	170 (27.9)	165 (25.7)	160 (25.1)	
Rural	1777 (73.5)	384 (72.5)	440 (72.1)	476 (74.3)	477 (74.9)	
Education level, *n* (%)						<0.001
Primary school or below	892 (36.9)	156 (29.4)	226 (37.0)	232 (36.2)	278 (43.6)	
Secondary school	1449 (59.9)	353 (66.6)	364 (59.7)	394 (61.5)	338 (53.1)	
College or above	77 (3.2)	21 (4.0)	20 (3.3)	15 (2.3)	21 (3.3)	
Income level, *n* (%)						0.281
Low	806 (33.3)	183 (34.5)	200 (32.8)	212 (33.1)	211 (33.1)	
Medium	808 (33.4)	158 (29.8)	212 (34.8)	233 (36.3)	205 (32.2)	
High	804 (33.3)	189 (35.7)	198 (32.5)	196 (30.6)	221 (34.7)	
Smoking status, *n* (%)						0.037
No	1802 (74.5)	372 (70.2)	452 (74.1)	485 (75.7)	493 (77.4)	
Yes	616 (25.5)	158 (29.8)	158 (25.9)	156 (24.3)	144 (22.6)	
Alcohol drinking status, *n* (%)						0.127
No	1683 (69.6)	353 (66.6)	426 (69.8)	440 (68.6)	464 (72.8)	
Yes	735 (30.4)	177 (33.4)	184 (30.2)	201 (31.4)	173 (27.2)	
Age, years, Median (IQR)	45.75 (38.39, 52.8)	45.75 (38.45, 52.69)	45.84 (38.18, 52.95)	45.85 (37.78, 52.76)	45.66 (39.32, 52.53)	0.990
BMI, kg/m^2^, Median (IQR)	22.55 (20.83, 24.77)	22.29 (20.73, 24.37)	22.41 (20.58, 24.59)	22.67 (20.76, 25.1)	22.89 (21.17, 24.85)	0.013
Sleep time, h/day, Median (IQR)	8 (7, 8)	8 (8, 8)	8 (8, 8)	8 (7, 8)	8 (7, 8)	0.144
Sedentary time, h/week, Median (IQR)	14 (10.5, 24)	14 (10.4, 24.38)	14 (9.67, 24.5)	14 (10.5, 23.33)	14 (10.25, 23)	0.895
Physical activity, MET-h/week, Median (IQR)	206.35 (105.35, 359.87)	198.88 (85.66, 334.34)	194.25 (106.66, 337.6)	210 (109.22, 359.9)	214.89 (110.7, 397.62)	0.038
Total energy intake, kcal/day, Median (IQR)	2167.37 (1789.13, 2638.3)	2227.47 (1819.92, 2682.24)	2234.21 (1855.86, 2660.75)	2113.64 (1788.8, 2610.36)	2111.53 (1701.6, 2562.5)	<0.001

Note: Dietary copper density was calculated as copper intake per 1000 kcal of total energy intake and expressed as mg/1000 kcal. Q1–Q4 were based on the unified quartile cutoffs used in the main analysis, which were derived from the pooled distribution of dietary copper intake at the start of the 2009–2015 and 2015–2018 intervals. Therefore, group sizes were not exactly equal. Continuous variables were compared using the Kruskal–Wallis rank-sum test, and categorical variables using the chi-square test.

**Table 2:** Incidence of individual components of MetS according to quartiles of energy-adjusted dietary copper intake.

Incidence	Overall	Q1	Q2	Q3	Q4	*p*_Value
Elevated blood pressure	961/1925 (49.9)	233/427 (54.6)	240/475 (50.5)	259/524 (49.4)	229/499 (45.9)	0.071
Elevated FPG	833/2335 (35.7)	230/505 (45.5)	211/594 (35.5)	184/625 (29.4)	208/611 (34.0)	0.366
Elevated TG	552/2044 (27.0)	130/449 (29.0)	139/514 (27.0)	132/543 (24.3)	151/538 (28.1)	0.813
Reduced HDL-C	594/2204 (27.0)	134/479 (28.0)	147/560 (26.3)	151/583 (25.9)	162/582 (27.8)	0.712
Abdominal obesity	599/2030 (29.5)	135/448 (30.1)	139/506 (27.5)	164/547 (30.0)	161/529 (30.4)	0.296

Note: *p* values were derived from the chi-square test comparing incidence proportions across quartiles of energy-adjusted dietary copper intake.

## Data Availability

Restrictions apply to the availability of these data. Data were obtained from our project group and are available (https://www.chinacdc.cn, accessed on 20 March 2026) with the permission of our project group.

## Abbreviations

The following abbreviations are used in this manuscript:

MetS	Metabolic Syndrome
CHNS	China Health and Nutrition Survey
RCS	Restricted cubic spline analysis
HDL-C	High-density Lipoprotein Cholesterol
BMI	Body Mass Index
